# Interaction of Nuclease Colicins with Membranes: Insertion Depth Correlates with Bilayer Perturbation

**DOI:** 10.1371/journal.pone.0046656

**Published:** 2012-09-28

**Authors:** Mireille Vankemmelbeke, Paul O′Shea, Richard James, Christopher N. Penfold

**Affiliations:** 1 School of Molecular Medical Sciences, Centre for Biomolecular Sciences, University of Nottingham, Nottingham, United Kingdom; 2 School of Biology, University of Nottingham, Nottingham, United Kingdom; Centre National de la Recherche Scientifique, Aix-Marseille Université, France

## Abstract

**Background:**

Protein transport across cellular membranes is an important aspect of toxin biology. *Escherichia coli* cell killing by nuclease colicins occurs through DNA (DNases) or RNA (RNases) hydrolysis and to this end their cytotoxic domains require transportation across two sets of membranes. In order to begin to unravel the molecular mechanisms underlying the membrane translocation of colicin nuclease domains, we have analysed the membrane association of four DNase domains (E9, a charge reduction E9 mutant, E8, and E7) and one ribosomal RNase domain (E3) using a biomembrane model system.

**Principal Results:**

We demonstrate, through the use of large unilamellar vesicles composed of synthetic and *E. coli* lipids and a membrane surface potential sensor, that the colicin nuclease domains bind anionic membranes only, with micromolar affinity and via a cooperative binding mechanism. The evaluation of the nuclease bilayer insertion depth, through a fluorescence quenching analysis using brominated lipids, indicates that the nucleases locate to differential regions in the bilayer. Colicin DNases target the interfacial region of the lipid bilayer, with the DNase E7 showing the deepest insertion, whereas the ribosomal RNase E3 penetrates into the hydrophobic core region of the bilayer. Furthermore, the membrane association of the DNase E7 and the ribosomal RNase E3 induces vesicle aggregation, lipid mixing and content leakage to a much larger extent than that of the other DNases analysed.

**Conclusions/Significance:**

Our results show, for the first time, that after the initial electrostatically driven membrane association, the pleiotropic membrane effects induced by colicin nuclease domains relate to their bilayer insertion depth and may be linked to their *in vivo* membrane translocation.

## Introduction

A recurrent theme in membrane biology is how water-soluble folded protein domains traverse the hydrophobic barrier posed by biomembranes. This is particularly important for protein toxins whose targets often reside in membrane-enclosed cellular compartments. They hence utilise a number of membrane translocation strategies such as co-opting protein-conducting translocons (as in the case of cholera, shiga and ricin toxins during their retrotranslocation from the endoplasmic reticulum into the cytosol [1]) or facilitating their own transport across the bilayer with (anthrax and diphtheria toxins [2]) or without (*Bordetella pertussis* CyaA toxin [3]) the help of a membrane-embedded facilitator.

Nuclease colicins, *E. coli* bacteriocins, are protein cytotoxins that kill closely-related Gram-negative species through DNA (DNases) or RNA (RNases) hydrolysis [4]. They consist of three functional domains associated with receptor binding, outer membrane (OM) translocation and cytotoxicity (nuclease domain). Their cytotoxic domains face a formidable challenge, crossing two membranes and the periplasm in order to reach their target inside the cytoplasm. Studies of the protein-protein interactions involved in their cellular uptake pathway have predominantly centred on OM translocation and have provided a wealth of information about the *E. coli* OM organisation and maintenance [5]. They have highlighted how nuclease colicins cunningly exploit OM receptors, porins and endogenous energised protein conduits to accomplish their final goal. In the case of the group A nuclease colicins this consists of engaging with the OM receptor BtuB, the porin OmpF and the periplasmic Tol system in order to bring about the release of their associated immunity proteins and concomitant OM translocation of the cytotoxic domains [6]–[10]. It has been suggested that immunity protein release from the cell-bound nuclease colicin complex causes unfolding of the cytotoxic domain thereby enabling it to utilise the OmpF channel for crossing the OM [11], [12]. More recently however, structural analysis of the receptor-bound complex of the pore-forming colicin N, which uses OmpF as its receptor and translocator, introduced an interesting new concept for OM translocation of colicin cytotoxic domains, suggesting that the bilayer-porin interface could be the site of membrane insertion which would enable the cytotoxic domain to bypass the central porin channel [13], [14].

The process of nuclease inner membrane (IM) translocation on the other hand has received considerably less attention. It is thought that a retrotranslocation event akin to that seen for certain other toxins that target eukaryotic cells is involved [1], [15]. Recognition of an IM-inserted colicin as a misfolded protein would thus trigger a dislocation event by a membrane-associated translocator. One potential candidate for this is the IM-associated AAA^+^ protease FtsH which has the unique ability to extract misfolded membrane proteins from the membrane for subsequent degradation [16]. This essential protein dislocator, normally involved in protein quality control through its proteolytic and chaperone-like activities, has been shown to be required for DNase translocation into the cytoplasm, requiring both its ATPase and protease activity [17]. Additionally, evidence for a proteolytic event linked with colicin import came from the use of cell extracts to produce a cleavage releasing the cytotoxic domains of colicins E7 and D [18]–[20]. More recently, this was further substantiated for two RNases, as was the involvement of FtsH, but the precise molecular events and how the nucleases escape degradation require elucidation [21]. So far however, attempts to show a direct interaction between FtsH and a nuclease colicin have been unsuccessful.

Colicin DNases and ribosomal RNases (rRNase) share a sequence rich in basic residues, in spite of their obvious structural differences. They also make use of an identical cellular uptake pathway and it is therefore likely that their membrane translocation events will be structure-independent processes. Furthermore, *in vitro* studies on the membrane interactions of the E9 and E3 nuclease domains revealed significant structural destabilisation as a result of their association with anionic lipids [22], [23]. There remains however some ambiguity regarding the degree of nuclease destabilisation *in vivo*, since the effects were studied with vesicles containing a high proportion of anionic lipids. Intrinsic channel forming activity of the colicin E9 DNase (and other DNase colicins) in planar lipid bilayer experiments has also been demonstrated, but this was not linked to cell killing [24]. The DNase-induced channel events were short-lived and not voltage-dependent in contrast to the channel activity of the pore-forming colicins that have much larger half-lives and kill cells through depolarisation of the inner membrane. More recently, the role for electrostatic attraction in nuclease membrane association was substantiated by data showing increased cell killing efficiency by DNase colicins carrying higher net positive charge, an effect which was more pronounced when a strain reduced in anionic phospholipid (PL) content was used [17]. In *E. coli*, PL are present in the inner leaflet of the OM (the outer leaflet consists of lipopolysaccharide) and in the IM and they comprise the neutral phosphatidylethanolamine (75%) and the anionic phosphatidylglycerol (20%) and cardiolipin (5%), the ratio of which is tightly regulated in order to maintain functionality [25]–[27]. It is therefore likely that the PL composition of biomembranes and their spatial organisation will affect *in vivo* nuclease translocation at the OM and IM.

It is currently unclear how the channel-forming activities of the DNase-type colicins are linked to their membrane translocation and why the rRNase E3 did not possess a similar activity in those experiments. Additionally, a detailed analysis of the effects of nuclease binding on membrane integrity and how this is governed by the physicochemical properties of the nucleases is currently lacking. In order to further our understanding of the molecular mechanisms underlying the membrane translocation of colicin nuclease domains, we have examined their membrane association *in vitro* using large unilamellar vesicles (LUVs) made up of synthetic lipids mimicking the *E. coli* PL charge ratio or an *E. coli* polar lipid extract. We have analysed the rRNase E3 and a number of DNases (E9 and a charge reduction E9 mutant (JB10), E8 and E7 (Table 1)), carrying a range of net positive charge [17], for their membrane binding affinity, bilayer insertion depth, and for the overall effect of their binding on the bilayer integrity and we present data that permit the assembly of a mechanistic translocation model.

**Table 1:** Nuclease colicins used in this work.

Construct	Number of residues	pI	Net positive charge	Hydrophobicity^b^
**DNase E7**	134	10.10	+13	195
**DNase E8**	134	9.90	+9	213
**DNase E9**	134	9.61	+7	205
**DNase JB10** a	134	9.02	+3	205
**rRNase E3**	96	9.73	+8	142

a DNase JB10 is an E9-based construct with two Lys residues (at positions 21 and 45) mutated to Glu [17].

b Hydrophobicity is calculated according to Hessa *et al*. where smaller values indicate larger hydrophobicity [28].

## Methods

### Constructs

Nuclease constructs and their properties are listed in Table 1. Nuclease hydrophobicity was calculated according to the ‘biological’ hydrophobicity scale of Hessa *et al.* which is derived from functional membrane integration assays [28]. Other hydrophobicity scales (Kyte and Doolittle [29] or Wimley and White [30]) show a similar hydrophobicity trend among the nucleases. Nuclease domains were expressed (pET21d, Novagen) as heterodimers with their cognate immunity proteins containing a C-terminal polyhistidine tag (His-tag) to facilitate purification from *E. coli* BL21(DE3) (Novagen).

### Protein purification and immunity protein removal

Nuclease complexes were purified as previously described by metal chelate chromatography [31]. Most immunity proteins were removed from the nuclease complexes (E9Im9, JB10Im9) via denaturing size-exclusion chromatography. Briefly, protein peaks from the initial purification were freeze-dried, solubilised in 100 mM sodium phosphate buffer containing 0.5 M NaCl and 50 mM citric acid pH 2.5 followed by separation of the immunity protein from the nuclease on a Superdex S-75 column (GE Healthcare) [32]. Cation-exchange chromatography on a mono-S column (GE Healthcare) using a 20 mM glycine buffer pH 3.0 was necessary in order to remove the immunity proteins from the DNase complexes E8Im8 and E7Im7. The free rRNase E3 was obtained from the column-bound His-tagged complex by elution with 6 M GnHCl. Fractions containing free nucleases were pooled, refolded by extensive dialysis against 10 mM potassium phosphate buffer (Kpi) pH 7.5 and stored.

### Vesicle preparations

All lipids were obtained from Avanti Polar Lipids. LUVs composed of 1,2-dioleoyl-sn-glycero-3-phosphocholine (DOPC) (75 mol %) and 1,2-dioleoyl-sn-glycero-3-phospho-(1′-rac-glycerol) (DOPG) (25 mol %) were prepared as previously described [33]. Briefly, lipids in chloroform were mixed and resuspended in 10 mM Kpi pH 7.5, after evaporation of the solvent. The mixture was vortexed extensively followed by five freeze-thaw cycles. Vesicles were sized (100 nm) via pressure-extrusion (10 x) through 0.1 µm polycarbonate filters and stored at 4°C in an oxygen-free atmosphere. Brominated LUVs were made in a similar manner with brominated phosphocholine ((6,7)-Br_2_-PC or (11,12)-Br_2_-PC) replacing DOPC. LUVs made from an *E. coli* polar lipid extract (*E. coli* B (ATCC 11303)) were also prepared as described above. Fluorescein phosphatidylethanolamine (FPE)-labelled LUVs were made by labelling the bilayer outer leaflet with FPE. Briefly, LUVs were incubated for 1 h with 0.1 mol % FPE dissolved in ethanol at 37°C in the dark. Unincorporated FPE was removed by gel filtration on a Sephadex G-25 column (GE Healthcare) equilibrated with the appropriate buffer. FPE-labelled vesicles were used within one week. Sulforhodamine B (SRB)-containing LUVs for the dye leakage analysis were made as described above but lipids (after solvent evaporation) were resuspended in 50 mM SRB (Invitrogen UK) in 10 mM Kpi pH 7.5. Unencapsulated dye was removed by gel filtration on a Sephadex G-25 column where SRB-containing LUVs were eluted in 10 mM Kpi pH 7.5, 0.1 M NaCl. Labelled vesicles for the FRET lipid mixing assay were prepared as above, but with the addition of 0.6 mol % N-(7-nitrobenz-2-oxa-1,3-diazol-4-yl)-1,2-dihexadecanoyl-sn-glycero-3-phosphoethanolamine (NBD-PE, donor) and 0.6 mol % N-(lissamine rhodamine B)-1,2-dihexadecanoyl-sn-glycero-3-phosphoethanolamine (Rh-PE, acceptor) both from Invitrogen, UK. For all fluorescence analyses, controls were performed to ensure the absence of scattered light contribution to the measured fluorescence signal.

### Steady-state analysis of nuclease domain interaction with FPE-LUVs

FPE-labelled vesicles in 10 mM Kpi pH 7.5 (200 µM lipid concentration) maintained at 25°C were subjected to serial additions of nuclease proteins in the same buffer (or buffer control) and FPE emission was monitored at 520 nm with 490 nm excitation using a FluoroMax®-4 Spectrofluorometer (Horiba Scientific). The data (F/F_0_, where F is the fluorescence intensity after each protein addition, averaged over 100 s, and F_0_ is the fluorescence intensity of the FPE-LUV suspension prior to protein addition) after subtraction of the buffer control, were fitted using non-linear regression to a hyperbolic or sigmoidal binding model using the GraphPad Prism 5 software package. The two models considered were: F = Fmax * [P]/(*K_D_*+[P]) (specific binding) and F = Fmax * [P] ˆ h/(*K_D_* ˆ h+[P] ˆ h) (specific binding with Hill slope) where F is the fluorescence variation, Fmax the maximum fluorescence variation, [P] the protein concentration, *K_D_* the dissociation constant of the membrane binding process and h the Hill coefficient indicating cooperativity. All nuclease titrations were performed at least twice with similar results.

### Brominated lipid quenching studies

Nucleases were incubated with brominated or non-brominated LUVs at a protein to lipid molar ratio (P∶L) of 1∶150 for 1 h at room temperature. Trp fluorescence was analysed on a Perkin-Elmer LS55 fluorescence spectrometer maintained at 25°C by a circulating water bath. An average of three scans was obtained with excitation at 280 nm and emission scanned between 300 to 400 nm. The percentage quenching was calculated using the following formula: % quenching = 1−(F/F_0_)×100 where F represents the fluorescence intensity in the presence of brominated LUVs and F_0_ with non-brominated LUVs.

### Vesicle aggregation assay

Protein-induced vesicle aggregation was monitored by following the changes in turbidity of a LUV suspension through monitoring the optical density (OD) at 436 nm. Protein aliquots were added to LUVs in 10 mM Kpi pH 7.5 (final lipid concentration, 100 µM) in a 1×1 cm cuvette. Absorbance was measured using a Varian Cary 100 Bio UV-Vis spectrophotometer. The temperature was maintained at 25°C. Results are presented as the increase in OD after protein addition compared to the vesicle suspension in the absence of protein.

### Membrane leakage analysis

SRB-containing LUVs of synthetic and *E. coli* lipids (SRB at self-quenching concentration) were incubated with the nuclease domains and the release of SRB monitored before and after nuclease addition with excitation at 565 nm and emission at 585 nm. The temperature was maintained at 25°C. The release was monitored for 5 min after which the addition of Triton X-100 (0.1% v/v) resulted in total release (100%). The percentage leakage was calculated as follows: % leakage = [F−F_0_/F_100_−F_0_]×100 where F is the fluorescence intensity 5 min after nuclease addition, F_0_ is the fluorescence intensity prior to nuclease addition and F_100_ is the fluorescence released by Triton X-100 addition. No background release was detected during the course of the experiments.

### Phospholipid-mixing FRET assay

Nuclease-induced vesicle lipid mixing was measured by resonance energy transfer [34]. This assay is based on the decrease in resonance energy transfer between two probes (NBD-PE and Rh-PE) when the lipids of the probe-containing vesicles are diluted through mixing with lipids from unlabelled vesicles. The concentration of each of the fluorescent probes was 0.6 mol %. Labelled and unlabelled vesicles in a 1∶4 ratio were incubated at a final lipid concentration of 100 µM at 25°C. Using 460 nm excitation, the emission was scanned from 500 to 650 nm and the ratio of the fluorescence at the donor (530 nm, F530) and acceptor (588 nm, F588) peaks taken before and after nuclease addition [35]. Since labelled and unlabelled vesicles were mixed in a proportion of 1∶4, 100% phospholipid mixing was deduced from a liposome preparation in which the concentration of each probe was 0.12 mol %. Phospholipid mixing was determined on a percentage basis according to the following equation: % mixing = [(R−R_0_)/(R_100_−R_0_)]×100 where R is F530/F588 after nuclease addition, R_0_ is the initial F530/F588 of the vesicles, and R_100_ is the F530/F588 value of the liposomes containing 0.12 mol % of each probe (representing 100% mixing) [35]. A buffer control was subtracted in each case.

## Results

### Colicin nuclease domains bind synthetic and E. coli lipid LUVs in a cooperative manner, with micromolar affinity

The electrostatic surface potential sensor, fluorescein phosphatidylethanolamine (FPE) has been used to great effect to monitor membrane association of a number of biologically important molecules such as viral fusion proteins, antimicrobial peptides (AMPs) and mitochondrial signal peptides [33], [36], [37]. We therefore labelled the outer leaflet of synthetic and *E. coli* lipid LUVs with FPE in order to assess the membrane binding affinity of the colicin nuclease domains listed in Table 1. The synthetic lipid LUVs were composed of 75 mol % DOPC and 25 mol % DOPG mimicking the *E. coli* PL charge composition. Additionally, LUVs made up from an *E. coli* polar lipid extract containing: cardiolipin (10%), phosphatidylglycerol (25%) and phosphatidylethanolamine (65%) according to the manufacturer's details were also analysed. Binding of the basic nuclease domains to FPE-labelled LUVs led to an increase in FPE fluorescence as a result of a change in its protonation state. Serial additions of the respective nucleases and plotting of the cumulative fluorescence change as a function of increasing nuclease concentration yielded binding curves that, after fitting to a hyperbolic or sigmoidal (cooperative) binding model, generated the equilibrium dissociation constants (*K_D_*) listed in Table 2. The *K_D_* values for the binding of the DNases to synthetic lipid LUVs range from 0.7 µM (E8) to 1.4 µM (JB10) with only small differences between the DNases. Although there appears to be a trend of increasing affinity with increasing net positive charge, with the exception of the DNase E7, this was not maintained for the *E. coli* lipid LUVs where *K_D_* values range from 1 µM (JB10) to 1.6 µM (E8) (Table 2). This suggests that *in vivo*, in the presence of a sufficiently negatively charged membrane surface, the increase in net positive charge of the DNases does not significantly affect their membrane affinity. Preliminary analysis of the nuclease membrane association dynamics using the same LUVs via stopped-flow fluorescence equally did not show a correlation between nuclease positive charge and the fast association rates (Vankemmelbeke & O′Shea unpublished data). It is likely however that a correlation between membrane affinity and nuclease net positive charge will become apparent when the negative charge of the bilayer is reduced. The rRNase E3 binds to the *E. coli* lipid LUVs with similar affinity as the DNase E9 and to the synthetic lipid LUVs with slightly lower affinity (Table 2).

**Table 2:** Equilibrium binding constants and Hill coefficients for the association of nuclease domains with synthetic or *E. coli* lipid LUVs.

	DOPC∶DOPG (3∶1)		*E. coli* lipids	
Construct	*K_D_* (µM)	Hill coefficient	*K_D_* (µM)	Hill coefficient
DNase E7	1.2±0.1	1.6±0.01	1.1±0.1	1.7±0.3
DNase E8	0.7±0.1	N.A.	1.6±0.2	N.A.
DNase E9	0.9±0.02	1.5±0.1	1.3±0.03	1.7±0.1
DNase JB10	1.4±0.2	1.4±0.02	1.0±0.1	1.1±0.01
rRNase E3	1.5±0.2	1.4±0.2	1.3±0.1	2.1±0.3

Incubations were performed in 10 mM Kpi, pH 7.5, at 25°C, with a lipid concentration of 200 µM. Standard errors are derived from the non-linear regression analysis. N.A. indicates absence of cooperativity. Nuclease titrations were carried out at least twice, with similar results.

Membrane association (synthetic and *E. coli* lipid LUVs) of most DNases and the rRNase E3 was best approximated by the sigmoidal binding model with Hill coefficients centring around 1.5 for the synthetic and 1.7 for the *E. coli* lipids (Table 2). This indicates the involvement of positive cooperativity in the nuclease membrane association. In the absence of a receptor protein, this could arise from multiple discrete interactions of distinct binding elements on the nucleases whereby a conformational rearrangement after the initial binding event leads to further more favourable interactions. This has been described as configurational cooperativity [38]. Alternatively, the positive cooperativity could be due to protein-protein interactions between membrane-bound nucleases. In this context, acidic pH-induced partial unfolding and subsequent multimerisation of the DNase E7 in solution have been demonstrated [39]. The DNase E8 is the only DNase whose binding profile is best approximated by a hyperbolic (non-cooperative) model. It is interesting to note that the apo-E8 DNase (in the absence of metal ions) has been shown to be more conformationally compact and thermostable than the other DNases, with the apo-DNase E7 being the most unstable [40]. This difference in stability and compactness among the DNases could explain the different binding models.

We also probed the full length colicin E9 for its membrane affinity and found that its *K_D_* and Hill coefficient are very similar to that of its isolated DNase domain (data not shown). This suggests that there is little or no contribution of the other domains to the membrane binding process. None of the nucleases bound to neutral LUVs (100 mol % DOPC), with the exception of the DNase E7 which bound very weakly (data not shown). This confirms the importance of electrostatic interactions in the membrane targeting process. Nuclease colicins are synthesised as high affinity heterodimeric complexes with their cognate immunity proteins in order to protect producing cells against suicide [41]. We therefore analysed the effect of immunity protein complexation on the nuclease membrane association and found that for DNases and the rRNase E3 alike, the presence of their cognate immunity protein completely abolishes their membrane association (data not shown). Charge neutralisation of the basic nucleases by the acidic immunity proteins is likely to account for this in view of the importance of electrostatic attraction in the initial membrane association. Additionally, it is possible that the immunity protein binding interface on the nucleases is precisely the region involved in their initial membrane targeting.

### Quenching analysis with brominated lipids reveals differential bilayer insertion depth of the nucleases

In order to further explore the membrane association of the nucleases, we analysed their bilayer insertion depth using brominated lipids. Quenching of tryptophan fluorescence by brominated lipids is exquisitely suited to probe the bilayer insertion depth of proteins [42]. Bromine atoms can be introduced in unsaturated acyl chains at the position of double bonds where they quench the fluorescence of nearby Trp residues. The mechanism of quenching is not entirely clear, but it has been postulated to be a combination of resonance energy transfer and collisional quenching, both of which are strictly distance dependent [42]. The colicin nucleases analysed in this study all contain two closely spaced Trp residues (all DNases at positions 22 and 58 and the E3 rRNase at positions 43 and 54), the fluorescence of which will be reduced when in close proximity to the bromine atoms in the hydrophobic core of the bilayer. Using DOPC-containing vesicles brominated at positions 6 and 7 along the acyl chain ((6,7)-Br_2_-PC, 75 mol %), we observed a large extent of Trp fluorescence quenching for the E3 rRNase, intermediate levels for the DNase E7 and a small, but significant amount for the DNases JB10, E9, and E8 (Table 3). No quenching was observed in the presence of cognate immunity proteins or 0.1 M NaCl (data not shown). The large extent of Trp fluorescence quenching for the rRNase E3 suggests that its Trp residues are less than 8 Å away from the bromine atoms in the hydrophobic core of the bilayer since the r_0_ for 50% quenching is 8 Å [42]. The intermediate and low levels of Trp fluorescence quenching for the DNases are an indication that their Trp residues reside in the interfacial region of the bilayer. This is a chemically heterogeneous region with a steep polarity profile which is often favored by amphipathic helical regions of peripheral membrane proteins [43]. Because of the large amounts of quenching observed for the rRNase E3 by (6,7)-Br_2_-PC, we also included LUVs containing (11,12)-Br_2_-PC (75 mol %) in our analysis. The E3 Trp fluorescence was quenched approximately 50% by (11,12)-Br_2_-PC (Table 3). The effective thickness of half a DOPC bilayer is approximately 28 Å [44] with the bromine atoms in (11,12)-Br_2_-PC positioned 6.3 Å from the centre [42]. This approximates the position of the E3 rRNase Trp residues to about 14.3 Å from the centre of the bilayer and indicates their insertion into the edge of the hydrophobic core of the bilayer. Additionally, the DNase E7 Trp residues also experienced some quenching (∼15%) by (11,12)-Br_2_-PC, suggesting they insert deeper than the Trp residues of the other DNases (E9, JB10 and E8) that experienced no quenching (Table 3).

**Table 3:** Percentage quenching of nuclease Trp fluorescence by brominated lipids.

Construct	(6, 7)-Br_2_-PC∶PG (3∶1) (%)	(11, 12)-Br_2_-PC∶PG (3∶1) (%)
**DNase E7**	23.7±3.7	14.7±1.9
**DNase E8**	5.2±2.2	N.Q.
**DNase E9**	12.3±1.3	N.Q.
**DNase JB10**	13.7±1.1	N.Q.
**rRNase E3**	85.6±2.9	48.7±6.6

Nucleases were incubated with LUVs at P∶L of 1∶150 in 10 mM Kpi pH 7.5. Standard errors are derived from the averaging of a minimum of three independent experiments. N.Q. denotes no quenching.

Bilayer insertion by membrane active peptides and proteins depends on the balance between electrostatic and hydrophobic interactions [45]–[47]. In the case of the nuclease domains, there appears to be a correlation between their hydrophobicity (Table 1) and the amount of quenching their Trp residues experience by (6,7)-Br_2_-PC (Table 3) such that E3>>E7>JB10≈E9>E8. We cannot rule out however, that the high net positive charge of the DNase E7 also contributes to its Trp residues inserting somewhat deeper than the other DNases. Cho & Stahelin have grouped peripheral membrane proteins according to their membrane location as S-type (superficial), I-type (interfacial, interacting with headgroups and hydrocarbons) and H-type (hydrocarbon core region) [48]. This would classify the DNases E8 as S/I-type, JB10 and E9 as I-types, E7 as I/H-type and the rRNase E3 as H-type. These results may be relevant to *in vivo* IM translocation of the nuclease domains where deeper bilayer penetration may facilitate access to a membrane-associated translocator such as FtsH.

### Nuclease binding induces pleiotropic membrane defects: evidence of vesicle aggregation, lipid mixing and content leakage

**Figure 1:**
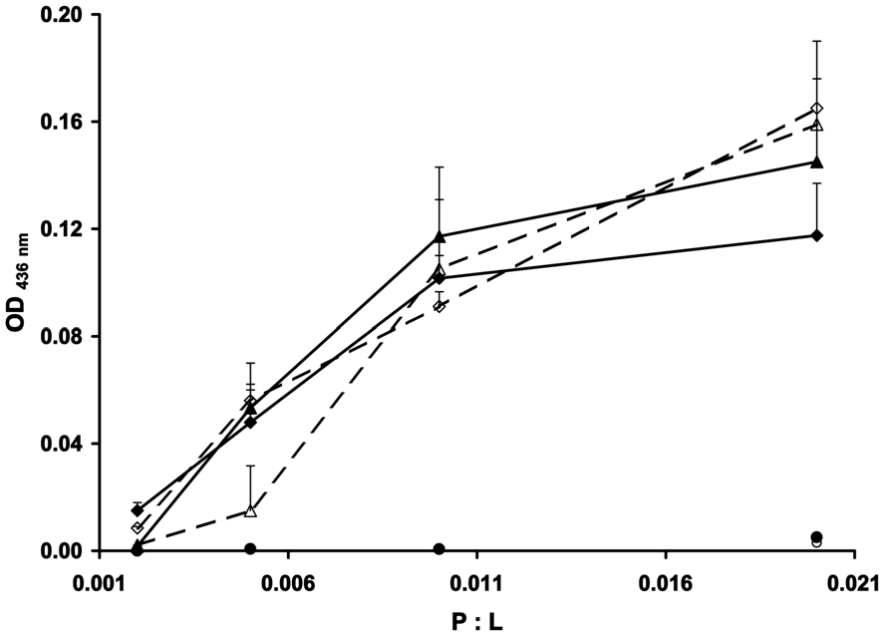
Effect of nuclease addition on LUV aggregation. Nuclease domains were added to a LUV suspension (100 µM lipid concentration) at a range of P∶L and the aggregation was monitored via measuring the OD at 436 nm. Nuclease addition to DOPC∶DOPG (3∶1) and *E. coli* lipid LUVs are depicted by closed and open symbols respectively. DNases: E7 (◊) and E9 (o), rRNase E3 (Δ). The DNases JB10 and E8 gave similar results to E9 and are omitted for clarity. The standard errors are derived from the averaging of a minimum of three independent experiments.

During the course of our experiments, we observed that some nucleases would cause an increase in turbidity when incubated with the acidic lipid containing LUVs (synthetic and *E. coli* extract). Protein-induced vesicle aggregation is an indication of close proximity of adjacent vesicles as charge neutralisation alleviates the electrostatic repulsion [49]. We analysed the nuclease-induced turbidity in LUV suspensions through monitoring the optical density at 436 nm over a range of P∶L [50]. The membrane association of the DNase E7 and the rRNase E3 leads to a concentration-dependent increase in vesicle aggregation (Fig. 1). At P∶L below 0.002 no aggregation is evident whereas at higher P∶L a rapid increase in optical density was observed, reaching a plateau after approximately two minutes. Vesicle aggregation by E7 and E3 is induced with similar efficiency for both the synthetic and *E. coli* lipid LUVs (Fig. 1). The sigmoidal relationship between E3 concentration and vesicle aggregation in the case of the *E. coli* lipid LUVs perhaps further strengthens the cooperative binding hypothesis for E3. The other DNases (JB10, E9 and E8) induce little or no vesicle aggregation at the P∶L that were analysed (Fig. 1). This was unanticipated for E8 and E9 who carry similar net positive charge as E3 and therefore could be expected to neutralise the acidic lipids upon binding. This suggests that the hydrophobic character of the nucleases also contributes to the aggregation process.

**Figure 2:**
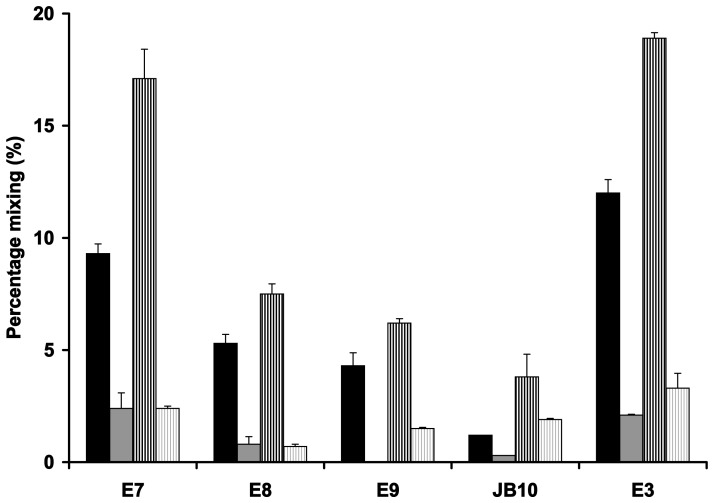
Extent of lipid mixing induced by nuclease membrane binding. Nuclease colicins were added at P∶L of 0.02 (black bars) and 0.002 (grey bars) to DOPC∶DOPG (3∶1, solid bars) or *E. coli* lipid (striped bars) LUVs. Lipid mixing was deduced from the loss of FRET efficiency after addition of the nucleases to a LUVs suspension containing NBD-PE- and Rh-PE-labelled (0.6 mol % each) LUVs with a four-fold excess of unlabelled LUVs. The standard errors are derived from the averaging of a minimum of three independent experiments.

Close apposition of lipid bilayers followed by lipid mixing are two key stages in membrane fusion. The vesicle aggregation results therefore encouraged us to explore the lipid mixing ability of the nucleases since turbidity analysis alone cannot discriminate between LUV aggregation and lipid mixing. We used a FRET-based assay whereby dilution of donor and acceptor probes through protein-induced lipid mixing of labelled and an excess of unlabelled LUVs leads to a reduction in FRET, usually monitored through an increase in donor fluorescence and/or a decrease in acceptor fluorescence [34]. In order to increase the sensitivity of the assay we used a ratiometric approach monitoring both the donor and acceptor fluorescence [35] as described in the Methods section. The two nucleases that have the highest LUV aggregative effect (the DNase E7 and rRNase E3) are also most proficient in lipid mixing, the degree of which increases with increasing P∶L and is similar for synthetic and *E. coli* lipids (Fig. 2). The other DNases (E8, E9 and JB10) also bring about a small amount of lipid mixing which is most pronounced at P∶L of 0.02. The small degree of lipid mixing in spite of the lack of detectable aggregation with those DNases is perhaps the result of the distinct sensitivity of both assays. It is likely that the inherent structural flexibility of the nucleases [51] contributes to their lipid mixing ability as has been shown for certain viral fusion proteins and AMPs [52], [53]. Although the overall amount of nuclease-induced lipid mixing is low, compared to bona-fide fusion proteins, it demonstrates the profound effect their binding has on bilayer organisation.

**Figure 3:**
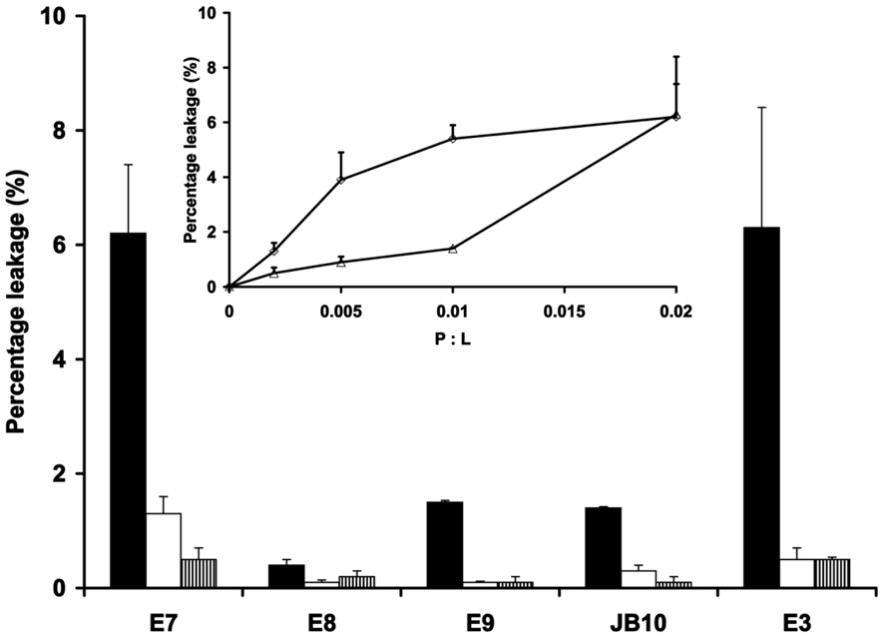
Vesicle leakage as a result of nuclease membrane binding. Nuclease colicins were added to SRB-containing LUVs; DOPC∶DOPG (3∶1) at P∶L of 0.02 (solid black bars) or 0.002 (solid white bars) and *E. coli* lipid at P∶L 0.02 only (striped bars). The SRB release after 5 min is presented as a percentage of total release (after Triton X-100 addition). The inset shows the effect of increasing nuclease (E7 (◊) and E3 (Δ)) concentration on the SRB release from DOPC∶DOPG (3∶1) LUVs. The standard errors are derived from the averaging of a minimum of three independent experiments.

Lipid mixing and membrane fusion often result in temporary defects in membrane integrity which can give rise to leakage of internal contents. We therefore analysed whether membrane association of the nuclease domains would lead to increased bilayer permeability using a dye leakage assay. SRB was encapsulated at self-quenching concentrations inside LUVs, the dilution of which in the external medium upon leakage gives rise to an increase in fluorescence which we monitored over a five-minute period. Colicin A, a true pore-forming colicin which kills sensitive *E. coli* cells via depolarisation of the inner membrane, was used as a positive control. At the P∶L of 0.02, all nucleases induce some content leakage from the synthetic lipid LUVs with the highest leakage observed for the E7 DNase and the E3 rRNase (Fig. 3). The percentage leakage induced by colicin A at this P∶L and under identical conditions is much higher (33%±1%), suggesting that the nuclease colicins affect membrane integrity via an altogether different mechanism. The rRNase E3 and the DNase E7 cause a dose-dependent increase in synthetic lipid LUV permeability, which shows a sigmoidal relationship with E3 concentration (Fig. 3, inset). Both nucleases induce leakage at a P∶L of 0.002 indicating that they affect bilayer integrity also at perhaps more physiologically relevant concentrations. The *E. coli* lipid vesicles were more refractory to leakage than the synthetic lipid LUVs and were therefore only analysed at the P∶L of 0.02 where a similar trend to the synthetic lipids was observed (Fig. 3). The pore-forming colicin A caused a release of 4%±1% from the *E. coli* lipid LUVs at this P∶L which is equally much lower than for the synthetic lipid LUVs. The large difference in content leakage from the synthetic lipid LUVs compared to the *E. coli* extract is likely to be a reflection of the different lipid composition and points to parameters such as membrane curvature and lipid phase behaviour affecting the process. No vesicle aggregation, lipid mixing and content leakage were observed in the presence of 0.1 M NaCl (data not shown), further emphasising the electrostatic nature of the initial nuclease membrane association.

The more pronounced vesicle content leakage by the E7 and E3 nucleases correlates well with their increased ability to cause vesicle aggregation and lipid mixing and with their deeper bilayer insertion compared to the other nucleases. The bilayer permeability data complement the earlier observations of ‘channel-like’ activity of DNase colicins in planar bilayer experiments [24]. It is intriguing that in those experiments no channel forming activity was detected with the rRNase E3 domain in spite of its effect on membrane permeability in our study. This perhaps suggests that the membrane destabilising effects observed for the rRNase E3 occur via a distinct mechanism compared to the DNases.

## Discussion

Many studies on the membrane actions of AMPs have led to a number of working models for their membrane disruptive behaviour and their structural arrangement within the membrane [46], [47], [54]. The current understanding points to the balance between electrostatic and hydrophobic interactions in the membrane environment determining the outcome of their membrane action [55]. Our data, presented here, show that this is also the case for the nuclease colicins, where long-range non-specific electrostatic interactions between the basic nucleases and the acidic headgroups (this work and [17]) drive the formation of the initial encounter complex which then facilitates further more specific interactions in such a way that their hydrophobicity determines their insertion depth and resulting bilayer destabilisation. Our results and previous work [22] demonstrate that *in vitro* physiological salt concentrations prevent the electrostatically driven membrane association of the nuclease domains. The *in vivo* relevance of those electrostatic attractions were further demonstrated via cell killing assays under conditions of reduced membrane charge by Walker *et al*. [17]. Biological activity of nuclease colicins however is not inhibited under physiological salt conditions suggesting that *in vivo*, other factors associated with biomembranes such as the presence of membrane proteins and lipid microdomains with, for instance, a high local net negative charge [56] are also affecting colicin membrane interactions.

The membrane perturbation caused by the nucleases is unlikely to involve transmembrane pore formation in view of the small extent of vesicle content leakage they induce compared to the pore-forming colicin A, but is probably the result of their effect on lipid packing and arrangement. A number of recent non-pore models (such as: ‘molecular shape’, ‘lipid clustering’, ‘sinking raft’ and ‘interfacial activity’) have attempted to explain this effect for AMPs, but they remain rather descriptive and offer no insight into the structural reorganisation of the lipid molecules resulting in increased permeability [55]. One thing they have in common is the fact that for membrane destabilisation to occur, the peptides need to work collaboratively which could fit with the cooperative binding mode we observed for most of the nucleases. Our *in vitro* cooperative binding model does not contradict the widely accepted single-hit killing mechanism of nuclease colicins *in vivo* since it is unlikely that all the membrane-bound colicin molecules are transported into the cytoplasm, a process postulated to involve an inner membrane-associated translocator. Additionally, whether there is a role for cooperativity *in vivo* will depend on the number of colicin molecules being held near the membrane surface.

It is apparent that the E3 and E7 nuclease-induced vesicle aggregation, lipid mixing and content leakage are correlated. Vesicle aggregates, presumably held together via membrane-bound protein bridges, allow close apposition of membranes and subsequent protein-induced lipid mixing and possibly fusion to occur [57]. It is tempting to speculate that the ‘fusogenic’ effect of those nuclease colicins is somehow linked to their membrane translocation as has been shown for a number of other toxins [58]–[60]. The recent low resolution structure of the colicin N-receptor complex [14], which shows the cytotoxic domain inserted at the lipid/porin interface, hence presents an attractive option for the nuclease colicins, since this arrangement would circumvent the need for threading the nuclease domains through the narrow OmpF pore. If the nuclease colicins follow this model, our results could be relevant to both OM and IM translocation and could also explain the superior killing efficiency previously observed for colicin E7 [17]. A similar scenario may also be the case for the rRNase E3. Alternatively, immunity protein release may cause sufficient unfolding of the nuclease domains enabling the use of OmpF for OM translocation [11], [12], in which case our results are still pertinent for their IM translocation.

It is unlikely that the nuclease colicins achieve IM translocation through ‘self-propulsion’, in spite of the documented intrinsic channel forming activity of the DNases [24]. Our analysis of the bilayer insertion depth of the DNases suggests that they target the interfacial region of the bilayer, whereas the rRNase with its increased hydrophobic character inserts into the top region of the hydrophobic core. It is possible that interactions between membrane-embedded domains of the nucleases and the IM-associated translocator FtsH initiate their dislocation; alternatively, the use of membrane-embedded chaperones such as the prohibitin homologues, HflK and HflC, or QmcA may enable the nucleases to gain access to FtsH, which has its ATPase and protease domains in the cytosol [61]. How recognition and translocation of the nucleases by FtsH is achieved, is currently unclear, but it is likely to be sequence-independent and it will be facilitated by the flexible nature of the nucleases and the structural destabilisation as a result of their interaction with the membrane. FtsH can initiate its dislocation and proteolysis from the N- and C-termini of substrates as well as from internal sites, with proteolysis being regulated by the degree of folding in such a way that tightly folded substrate domains stop further degradation due to the weak unfolding activity of FtsH [61]. This may be one way in which nuclease domains escape proteolysis and initiate cell killing, by their rapid refolding in the cytoplasm.

Although the three dimensional structure and the relative contribution of secondary structural elements of the DNases and the rRNase in this study are clearly distinct, they share physicochemical properties, such as their cationic and amphipathic character [62], that predispose them to interact with and disrupt membranes in their quest to gain access to their cytoplasmic target. We identified at least three amphipathic helical segments with high hydrophobic moments and intermediate hydrophobicities within the DNase domains with the Heliquest algorithm [63] which groups them at the boundary between surface seeking and membrane penetrating helices [64], where their combined effect could contribute to the overall affinity and membrane destabilisation observed for the DNases. In the case of the rRNase E3, a number of its core beta strands possess a similar amphipathic character. The cooperative nature of the nuclease membrane association, demonstrated in this work, is equally evident for both the DNases and the rRNase. This perhaps suggests that the initial membrane association triggers a conformational rearrangement to allow further interactions [38]. This sequence of events ties in with the structural destabilisation of the nuclease domains caused by their association with acidic model membranes [22], [23] and stopped-flow data demonstrating multiphasic kinetics for the nuclease membrane association (Vankemmelbeke and O′Shea, unpublished data).

The membranotropic actions of the rRNase E3 show many similarities with those of the fungal ribotoxin alpha-sarcin with which it shares a composition rich in beta-structure [65], [66]. Alpha-sarcin contains three discrete membrane interaction regions, one of which, the N-terminal beta hairpin, is not only required to target specific ribosomal proteins, but is also the region where many of the electrostatic interactions with the acidic membrane surface take place [67]. It is tempting to speculate that the N-terminal immunity protein binding site of the rRNase E3 which is also involved in ribosomal protein binding [68], is equally important in the membrane targeting process, given its basic nature, amphipathic character, inherent flexibility, lack of interactions with the main beta sheet and absence of membrane binding in the presence of immunity protein. The same could hold true for the DNase immunity protein binding region which, in addition, is also the least conserved part of the DNases and could thus explain the observed differences in their membrane activities.

We have previously put forward the hypothesis [7] that colicin cell entry may only occur at specialised sites across the cell surface similar to phage uptake [69]. These ‘competent’ sites could comprise sites of close apposition of outer and inner membrane (‘so called Bayer's junctions’) and/or regions of local high curvature; the latter of which is now increasingly recognised as a geometric cue for protein localisation [70]. The amphipathic motifs within the nuclease domains may be one way in which they achieve recognition of highly curved membranes [71]. Both features (membrane apposition and curvature) may increase nuclease translocation efficiency in a number of ways. The greater membrane tension at sites of high curvature may facilitate protein insertion whereas membrane apposition could limit the need for periplasmic chaperones and could enable the coordinated translocation across both membranes.

Bacteriocins are currently showing enormous potential for biotechnological and biomedical applications [72]–[74]. For their successful exploitation, it is fundamental to first understand how they breach the bacterial membrane defence systems. Our data on the membrane perturbing actions of nuclease colicins offer an insight into this and future work will consist of topological and mechanistic investigations of the nuclease membrane translocation process.
